# Amalgam Fillings in Teeth

**Published:** 1899-06

**Authors:** Wm. L. Morgan

**Affiliations:** Baltimore, Md.


### AMALGAM FILLINGS IN TEETH.

Wm. L. Morgan, M. D., Baltimore, Md.

Mrs. W., age about thirty, tall, slender, dark hair and eyes, sallow complexion; generally unhealthy appearance; enlarged indurated submaxillary glands. Some years ago had had an enlarged tumor of same kind taken from right side of neck, back and below the ear; also suffering from indigestion, headache, neuralgia of face and jaws. All symptoms indicating Mercury.

Patient especially desired to get rid of the tumors. My impression is that Mercury was the first remedy given, but afterwards from the broad, flabby, indentated tongue, and other symptoms, gave Hepar Sulphur, which slightly improved the digestion and relieved the headaches. She had many large amalgam fillings in the teeth and a red rubber plate. I assured her they were the cause of her trouble. After some months' treatment without further benefit, she consented to have them removed, and gold fillings and a black rubber plate replaced the former fillings. Then Merc-viv., CM, one dose, and S. L. was given. Within three weeks the enlarged glands were visibly diminished, health and complexion much improved. Six weeks later enlargement of glands diminished by half; complexion and health good; the lady well pleased and glands still diminishing at last interview.

With three months' more treatment the enlargement of the glands will be entirely gone. The last interview was about a week ago.

This is given from memory, and dates not remembered.

### DISCUSSION.

Dr. Pease—The real interest in this paper hinges of course upon the causative influence of the Amalgams. I think that in cases like this we should credit the Amalgam with the symptoms that were so obstinate and persistent. There is no doubt that the red vulcanite had its influences also, and that the two substances, Amalgam and red vulcanite are allied in producing the effects in such cases, because both of them contain at least one of the preparations of Mercury. I have here some clinical notes and provings of Amalgam, which I have verified in practice. These are all characteristics of the effect of Amalgam: First, ulceration of the edges of the tongue, of the gums and buccal membranes; second, chronic bad breath. Now, that breath is peculiar. It is not like the breath of a syphilitic patient, nor breath from ozena, or ulcerated nasal passages. I cannot describe it. Imprints of teeth, which is a general Mercury symptom. Headache is general, with neuralgic pains in the sides of the face. For example, the patient will suffer from a disagreeable painful headache, and associated with it are twinges of pain which resembles that which they have in the head. It will go into the sides of the face, and is felt in the teeth and in the ears. Pains in the feet, worse in hot and rainy weather. Dysenteric attacks with the characteristic mercurial painful stools, and these dysenteric attacks will come again and again. They do not run their regular course. A patient may suffer with an attack of dysentery, which will last perhaps twenty-four hours or it may last several days, but the twenty-four hour attack will be just as bad as the one which lasts two or three days. Periodical attacks of tonsilitis attended with great thirst, slow development of pus, fetid breath, much dryness of the throat and buccal cavity, or profuse saliva with sensations of dryness. Inability to swallow food; it may be for days. Attacks suddenly grow worse at night, and more often on the left side. There are marked changes in the quality of voice, especially in singers. This I have verified.

Now in connection with this subject I will give the salient points in the case of a young lady twenty-three years of age, dark hair and eyes, robust, well-developed figure. For several years she had suffered from repeated and periodical attacks of tonsilitis. The attacks would come on suddenly and at the same time of year. She would go to bed feeling usually well, and awake in the night or in the morning with a fully developed attack of tonsilitis. Pain, swelling, inability to swallow food, and having great dryness of the mouth and throat. She had received all manner of remedies for the attacks during the several years, and at last a specialist removed the tonsils. Her singing voice was a deep, full, and musical contralto. About six months before her tonsils were removed her voice changed until it was much less musical, much deeper tones and with inclination to hoarseness. She was very much alarmed because her professional engagements were interfered with. After the tonsils were removed the voice did not improve at all, in fact, became more husky and coarse in timbre. This condition continued for something like two years following the tonsilotomy, and she also had attacks of this same tonsilitis without the sore tonsils. The attacks would involve the entire field—the soft palate, larnyx, etc. She came under my observation about last February. I took very carefully the history of her case. She had been under the care of a good homœopath with negative results, since very soon after the tonsils were removed. When the case came into my hands, knowing that a good prescriber had done good work, and recognizing the symptoms that had disappeared under the potentized Amalgam, I concluded to try potentized Amalgam. I ordered the removal of all the Amalgam fillings from her teeth. She promised that this should be done. I gave Amalgam, CM. Some days afterwards the patient came to my office and told me that she had had a severe attack of tonsilitis, that it only lasted two days, and that she had noticed that her voice had changed very much in the succeeding seven days. Her appetite had returned. She felt very different from what she had following the previous attacks. I gave Bro. Kennedy's "next best remedy," and in two months her voice had returned to its original pitch, the "husky coarseness" had disappeared, and the voice had regained its original purity of tone. She returned to health, and I have not had to prescribe for that case since.

Dr. Kennedy—How long after you gave the dose of Amalgam were the fillings removed?

Dr. Pease—About three weeks.

Dr. Patch—Have you ever had a case of that kind when the fillings were not removed, and given the Amalgam?

Dr. Pease—Yes, sir. In a case of recurring dysentery with the ulcerations in the mouth. With the potentiated Amalgam I could only palliate the case. They would return but not so severe. Then the amalgams were removed. There were no amalgams left in the mouth to all appearances. Still the attacks would return. Pains in the feet, especially in the heels. I examined the mouth one day very carefully, and buried in the gums were the remains of a tooth. I ordered it removed, and in it was found quite a large filling of amalgam, which had been hidden for years. The case recovered after that.

Dr. Close—Theoretically, it seems as though the susceptibility of the individual to the amalgam filling should be removed by a high potency, even with the old filling remaining, because it is largely a question of susceptibility.

Dr. Pease—I can say this, Dr. Close, that in several cases where the patients have absolutely refused to have the amalgams removed because they could not afford it, the conditions have disappeared under the potentized amalgam, and have not returned. I can safely say there are several cases where the amalgam symptoms have been removed, and yet the amalgam remained in the teeth.

Dr. Adams—My experience has been that while the dose of potentized vulcanite relieved temporarily, as long as the plate remained the attacks remained. Finally changing to black rubber was followed by intense aggravation in one case, but after that went away the patient was better than for some years.

Dr. Pease—The mercurial miasm is different with different individuals. There is another point to think of in connection with these cases. If the potency does not remove the susceptibility to amalgam, it is probable that the organism of that patient suffers from some other form of mercurial disease, to which Amalgam is not the simillimum?

Dr. Close—There are yet other points to consider. One is that dentists use different kinds of amalgam. Another point is that Mercury in its various forms has been used a much longer time than Iodine and many other drugs have, and the hereditary element enters in. It is an older and therefore a more obstinate miasm.

Dr. Pease—That difference in the various amalgams is an important point. The difficulty in producing the required results with only one potency of the amalgam might be to a great degree lessened by making a compound of many different amalgams, and potentiating it.

Dr. Young has just asked me if in cases where I have observed both gold and amalgam fillings, there have been any different symptoms. I believe I have noticed quite a difference in systemic symptoms. Patients who have both are much more subject to neuralgia without inflammation than are those with amalgam fillings only.

Dr. Morgan—I have made the same observation.

Dr. Pease—Can there be in these cases a possible battery current between the gold and amalgam, with the saliva acting as the battery fluid?

